# DeepSeek-R1 outperforms Gemini 2.0 Pro, OpenAI o1, and o3-mini in bilingual complex ophthalmology reasoning

**DOI:** 10.1016/j.aopr.2025.05.001

**Published:** 2025-05-09

**Authors:** Pusheng Xu, Yue Wu, Kai Jin, Xiaolan Chen, Mingguang He, Danli Shi

**Affiliations:** a School of Optometry, The Hong Kong Polytechnic University, Kowloon, Hong Kong, China; b Eye Center, The Second Affiliated Hospital of Zhejiang University School of Medicine, Hangzhou, China; c Research Centre for SHARP Vision (RCSV), The Hong Kong Polytechnic University, Kowloon, Hong Kong, China; d Centre for Eye and Vision Research (CEVR), 17W Hong Kong Science Park, Hong Kong, China

**Keywords:** Large language models, DeepSeek, Gemini, OpenAI, Clinical decision support, Reasoning ability, Ophthalmology professional examination

## Abstract

**Purpose:**

To evaluate the accuracy and reasoning ability of DeepSeek-R1 and three recently released large language models (LLMs) in bilingual complex ophthalmology cases.

**Methods:**

A total of 130 multiple-choice questions (MCQs) related to diagnosis (n ​= ​39) and management (n ​= ​91) were collected from the Chinese ophthalmology senior professional title examination and categorized into six topics. These MCQs were translated into English. Responses from DeepSeek-R1, Gemini 2.0 Pro, OpenAI o1 and o3-mini were generated under default configurations between February 15 and February 20, 2025. Accuracy was calculated as the proportion of correctly answered questions, with omissions and extra answers considered incorrect. Reasoning ability was evaluated through analyzing reasoning logic and the causes of reasoning errors.

**Results:**

DeepSeek-R1 demonstrated the highest overall accuracy, achieving 0.862 in Chinese MCQs and 0.808 in English MCQs. Gemini 2.0 Pro, OpenAI o1, and OpenAI o3-mini attained accuracies of 0.715, 0.685, and 0.692 in Chinese MCQs (all *P* ​<0.001 compared with DeepSeek-R1), and 0.746 (*P* ​= ​0.115), 0.723 (*P* ​= ​0.027), and 0.577 (*P* ​<0.001) in English MCQs, respectively. DeepSeek-R1 achieved the highest accuracy across five topics in both Chinese and English MCQs. It also excelled in management questions conducted in Chinese (all *P* ​<0.05). Reasoning ability analysis showed that the four LLMs shared similar reasoning logic. Ignoring key positive history, ignoring key positive signs, misinterpretation of medical data, and overuse of non–first-line interventions were the most common causes of reasoning errors.

**Conclusions:**

DeepSeek-R1 demonstrated superior performance in bilingual complex ophthalmology reasoning tasks than three state-of-the-art LLMs. These findings highlight the potential of advanced LLMs to assist in clinical decision-making and suggest a framework for evaluating reasoning capabilities.

## 1 Introduction

Large language models (LLMs), such as OpenAI's GPT series^1^ and Google's Gemini series,^2^^,^^3^ have revolutionized the field of artificial intelligence (AI) by demonstrating impressive capabilities in natural language understanding and reasoning. These models exhibit significant potential in the medical domain, including personalized health consultations, research and clinical decision support, surgical planning assistance, and the facilitation of telemedicine.^4^ However, their performance and safety must undergo rigorous evaluation before they can be responsibly integrated into clinical workflows.^5^

In ophthalmology, researchers have tried to use the LLMs to integrate massive amounts of ophthalmic medical literature, guidelines, and patients' data to assist doctors in making more accurate diagnoses and facilitating clinical decision support.^6^, ^7^, ^8^, ^9^, ^10^, ^11^, ^12^ However, existing LLMs have not yet meet the rigorous standards required for clinical adoption in ophthalmic disease diagnosis.^13^ For example, Bahir et al. found that Gemini Advanced only got a 66% accuracy rate in an ophthalmology residency exam.^14^ Similarly, Zhang et al. found that GPT-4o exhibited significantly lower accuracy in primary diagnosis compared to human ophthalmologists in twenty-six glaucoma cases.^15^ Notably, while LLMs may perform well on recall tasks, they face challenges when handling complex medical cases that require reasoning. For example, GPT-4o achieved mean accuracies of only 48.0% and 63.0% in diagnosing and determining the next step in reasoning tasks derived from JAMA Ophthalmology's Clinical Challenges section.^16^

Recently, the DeepSeek team released its latest cost-effective open-source model, DeepSeek-R1.^17^ By incorporating multi-stage training and cold-start data prior to large-scale reinforcement learning (RL), DeepSeek-R1 achieved performance on reasoning tasks comparable to OpenAI-o1-1217. However, its accuracy and clinical applicability in complex ophthalmology reasoning tasks, particularly in a bilingual context, remain uncertain. Bilingual capabilities are essential in clinical ophthalmology, as medical professionals often need to interpret patient records, guidelines, and research findings across multiple languages, especially in multilingual regions or international collaborations. Accurate language comprehension is critical for reducing misinterpretations and ensuring precise diagnoses and treatment recommendations.

This study aims to evaluate the performance of state-of-the-art (SOTA) reasoning LLMs, including DeepSeek-R1, Gemini 2.0 Pro, OpenAI o1, and OpenAI o3-mini, in bilingual complex ophthalmology reasoning. By assessing their accuracy and reasoning ability, we seek to determine their potential for real-world clinical applications and identify areas for future improvement.

## 2 Methods

### 2.1 Data sources

**Fig. 1:**
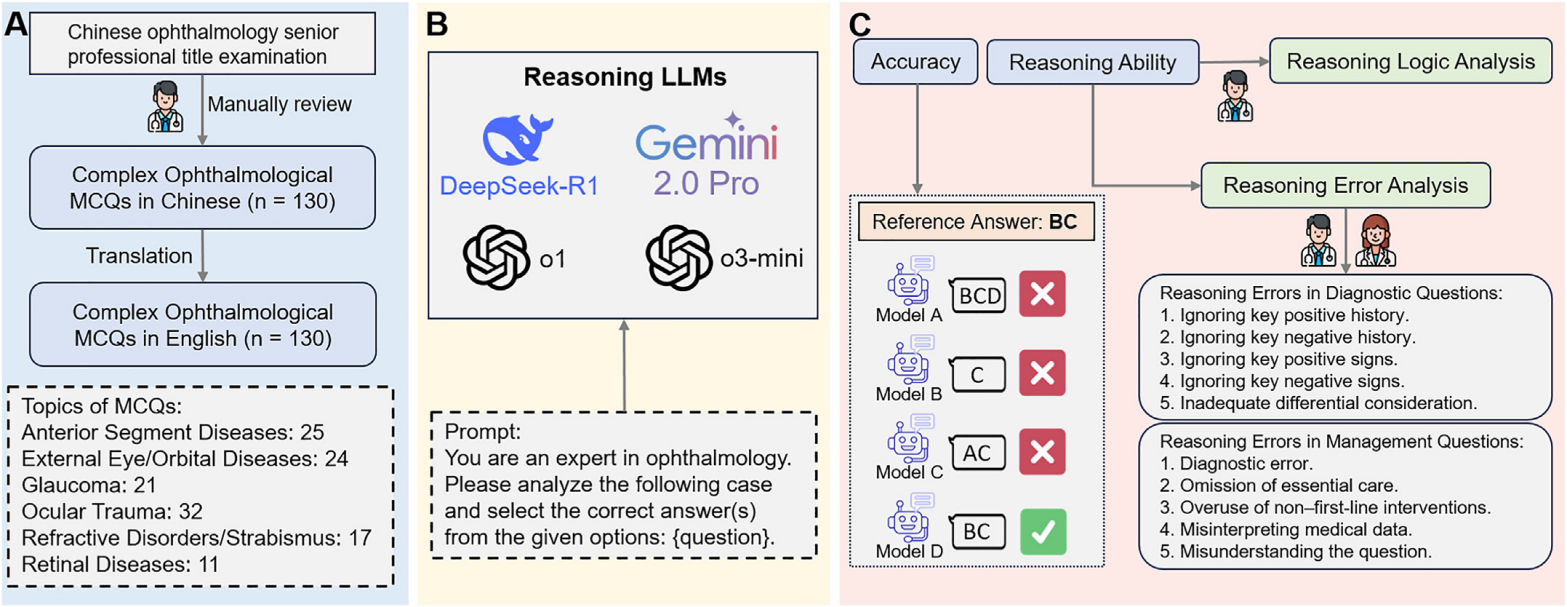
Overview of the study. MCQs ​= ​multiple-choice questions. LLMs ​= ​large language models. (A) Bilingual MCQs Preparation, (B) Get Responses from Reasoning LLMs, (C) Performance Evaluation.

To prevent potential data leakage—where test data is used in model training—we didn't use the publicly accessible USMLE questions that had been utilized in previous studies.^18^^,^^19^ Instead, we collected 130 multiple-choice questions (MCQs) designed for the Chinese ophthalmology senior professional title examination from VIP documents on Baidu Wenku. These MCQs were reviewed for validity and reliability by an ophthalmologist with over six years of clinical experience. The questions assess diagnostic (including differential diagnosis, n ​= ​39) and management (n ​= ​91) aspects across various ophthalmic subspecialties. We categorized them into six main topics: anterior segment diseases (n ​= ​25), external eye/orbital diseases (n ​= ​24), glaucoma (n ​= ​21), ocular trauma (n ​= ​32), refractive disorders/strabismus (n ​= ​17), and retinal diseases (n ​= ​11). Each question contains 5 to 9 answer choices, with the number of correct answers ranging from 1 to 6. An overview of this study is presented in Fig. 1.

### 2.2 Translation of MCQs

Considering that the same question in different languages may affect the performance of LLMs.^20^ We used DeepSeek-R1 to generate the English version of these MCQs. To mitigate the risk of the model unintentionally memorizing the correct answers during translation, only the questions and answer options were input, excluding the reference answers. The prompt used for this translation process is provided in Supplemental Table 1A. All translated MCQs were manually reviewed by an ophthalmologist with over six years of clinical experience to ensure accuracy and medical relevance.

### 2.3 LLM access

We accessed DeepSeek-R1 via the Application Programming Interface (API) provided by Volcengine, a cloud service platform under ByteDance, as the official DeepSeek server has been experiencing attacks, overload pressure, and usage limitations. Gemini 2.0 Pro (Gemini-2.0-pro-exp) and OpenAI o3-mini were accessed through their official APIs. Due to OpenAI's restrictions on API access for o1, we were unable to retrieve responses via the official API and instead obtained them through the official chat user interface (UI).

To ensure linguistic consistency between the responses and the corresponding MCQs, we used prompts aligned with the language of the questions (Supplemental Table 1B and 1C). Each LLM was tested using the same fixed sequence of questions in a single testing round. All responses were generated under default configurations between February 15 and February 20, 2025.

### 2.4 Reasoning ability analysis

Complex reasoning in medicine is a process of integrating multiple sources of clinical information, including patient history, physical examination findings, diagnostic imaging and laboratory results, and clinical knowledge, through a multistep logical framework to reach an accurate diagnosis or an appropriate management plan. In this study, reasoning ability was assessed by analyzing reasoning logic and identifying the causes of reasoning errors. The analysis of reasoning logic involved examining and comparing the reasoning processes used to answer questions correctly across all models. The causes of reasoning errors in both Chinese and English responses from LLMs were independently analyzed by two ophthalmologists (P.X. and Y.W.), each with 3–6 years of clinical experience. Interrater reliability was determined using Cohen's kappa. The categories of reasoning errors were predefined through pilot testing of 60 MCQs to identify error patterns. For diagnostic-related questions, errors were classified into five categories: 1. Ignoring key positive history. 2. Ignoring key negative history. 3. Ignoring key positive signs. 4. Ignoring key negative signs. 5. Inadequate differential consideration. For management-related questions, errors were also classified into five categories: 1. Diagnostic error, which means the answer was chosen based on an incorrect diagnosis. 2. Omission of essential care, which means failing to suggest necessary diagnostic or therapeutic interventions, potentially resulting in delayed or suboptimal management. For example, ignoring the role of adjunctive therapy or recommending conservative treatment when surgical intervention is indicated. 3. Overuse of non–first-line interventions. For example, recommending a more expensive or higher-risk test or treatment when a cheaper or safer alternative is available. 4. Misinterpreting medical data. For example, misinterpreting disease characteristics and complications, or misunderstanding the indications and contraindications of medications or surgeries. 5. Misunderstanding the question. For example, providing multiple answers when the question asks for the most important or most urgent action. The categorization was based on established clinical practice guidelines and consensus statements that are broadly recognized internationally, such as those from the American Academy of Ophthalmology (AAO) and relevant international societies.

### 2.5 Statistical analysis

The final answers chosen by the LLMs were manually verified based on the text responses of the models. Accuracy was calculated as the ratio of correctly answered questions to the total number of questions. Since some MCQs contained multiple correct answers, both omitted and extra answers were considered incorrect in this study. A 95% confidence interval was calculated using the Clopper-Pearson method. P-values were computed using McNemar's test, with *P* ​<0.05 considered statistically significant. Statistical analyses were performed using Stata/MP 17.0 (StataCorp, College Station, TX, USA). Radar charts, grouped bar charts and stacked bar charts were created with Origin 2025 (OriginLab Corporation, Northampton, MA, USA).

## 3 Results

### 3.1 Overall performance of the four LLMs

As shown in Table 1A, DeepSeek-R1 demonstrated a leading performance in Chinese complex ophthalmology reasoning tasks, achieving an overall accuracy of 0.862 (95%CI: 0.790–0.916; all *P* ​<0.001 when compared with three other LLMs). Gemini 2.0 Pro ranked second, with an overall accuracy of 0.715 (95%CI: 0.630–0.791); however, its performance was comparable to OpenAI o1 and o3-mini, as no statistically significant differences were observed.

**Table 1:** | Overall accuracy of DeepSeek-R1 and three other Large Language Models in Chinese (A) and English (B) complex ophthalmology reasoning.

A			
Models	Accuracy	95%CI	*P* value
**DeepSeek-R1**	**0.862**	[0.790, 0.916]	–
**Gemini 2.0 Pro**	0.715	[0.630, 0.791]	<0.001^a^
**OpenAI o1**	0.685	[0.597, 0.763]	<0.001^a^
**OpenAI o3-mini**	0.692	[0.605, 0.770]	<0.001^a^

B			
Models	Accuracy	95%CI	*P* value
**DeepSeek-R1**	**0.808**	[0.729, 0.872]	–
**Gemini 2.0 Pro**	0.746	[0.662, 0.818]	0.115
**OpenAI o1**	0.723	[0.638, 0.798]	0.027
**OpenAI o3-mini**	0.577	[0.487, 0.663]	<0.001^a^

a Denotes statistically significant for comparisons against DeepSeek-R1's performance. CI ​= ​confidence interval.

Although DeepSeek-R1 performed lower accuracy in English reasoning tasks than in Chinese (Table 1B), it still ranked first, achieving an overall accuracy of 0.808 (95%CI: 0.729–0.872; *P* ​= ​0.115, 0.027 and ​<0.001 when compared with Gemini 2.0 Pro, OpenAI o1 and o3-mini, respectively). Additionally, both Gemini 2.0 Pro and OpenAI o1 exhibited higher accuracy in English reasoning tasks. In contrast, OpenAI o3-mini demonstrated worse performance in English (*P* ​= ​0.017, Supplemental Table 2), with an accuracy of only 0.577 (95%CI: 0.487–0.633), placing it in fourth position.

### 3.2 Performance of LLMs in different topics

**Fig. 2:**
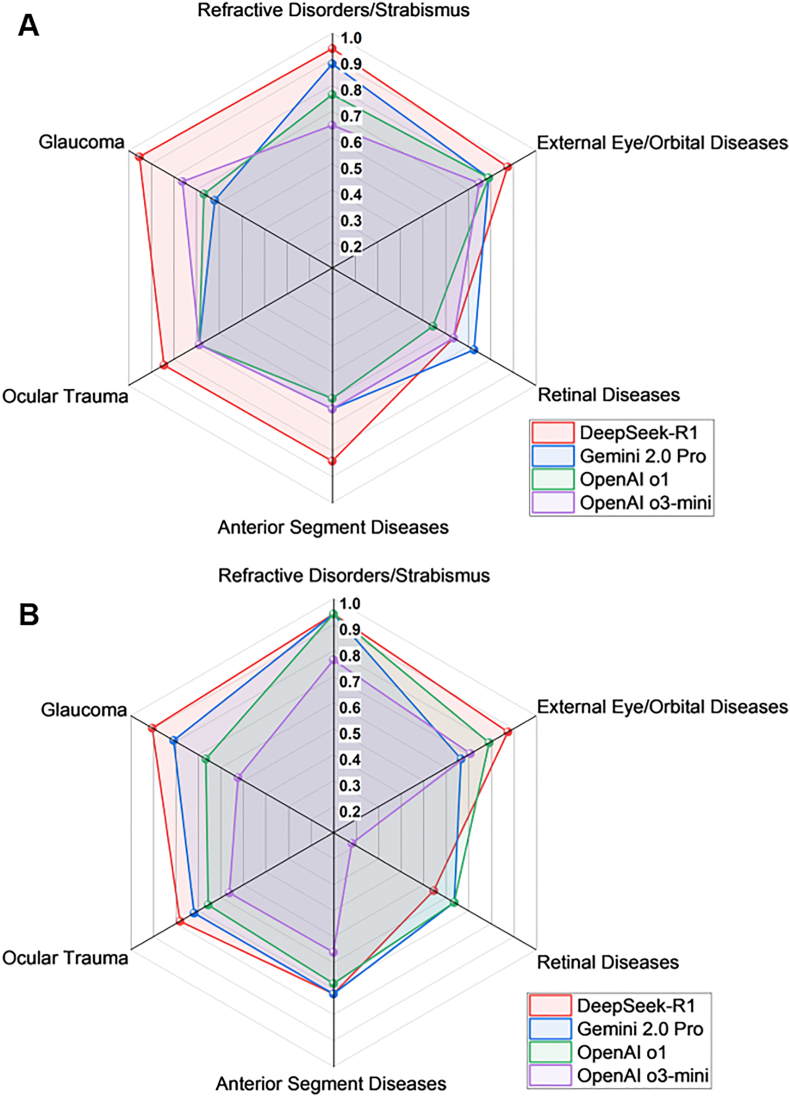
Radar charts depicting the performance of the four LLMs across different topics. (A) Chinese multiple-choice questions, (B) English multiple-choice questions.

In the Chinese MCQs, DeepSeek-R1 achieved the highest accuracy in five topics (Fig. 2A), including glaucoma (0.952, 95%CI: 0.762–0.999), refractive disorders/strabismus (0.941, 95%CI: 0.713–0.999), external eye/orbital diseases (0.875, 95%CI: 0.676–0.973), ocular trauma (0.843, 95%CI:0.672–0.947), and anterior segment diseases (0.840, 95%CI: 0.639–0.955). However, only statistically significant when compared with Gemini 2.0 Pro in the topic of glaucoma (Supplemental Table 3A). Gemini 2.0 Pro achieved the highest accuracy in retinal disease topic with an accuracy of 0.727 (95%CI: 0.390–0.940).

In the English MCQs, DeepSeek-R1 also ranked first in five topics (Fig. 2B): refractive disorders/strabismus (0.941, 95%CI: 0.713–0.999), glaucoma (0.905, 95%CI: 0.696–0.988), external eye/orbital diseases (0.875, 95%CI: 0.676–0.973), ocular trauma (0.781, 95%CI: 0.600–0.907), and anterior segment diseases (0.720, 95%CI: 0.506–0.879). However, statistical significance was only observed when compared with OpenAI o3-mini in the topics of glaucoma and ocular trauma (Supplemental Table 3B). Gemini 2.0 Pro shared the highest accuracy in three topics, while OpenAI o1 shared the highest accuracy in two topics. All models exhibited poor performance in retinal diseases, with OpenAI o3-mini achieving the lowest accuracy of 0.182 (95%CI: 0.023–0.518).

### 3.3 Performance of LLMs in diagnostic and management questions

**Fig. 3:**
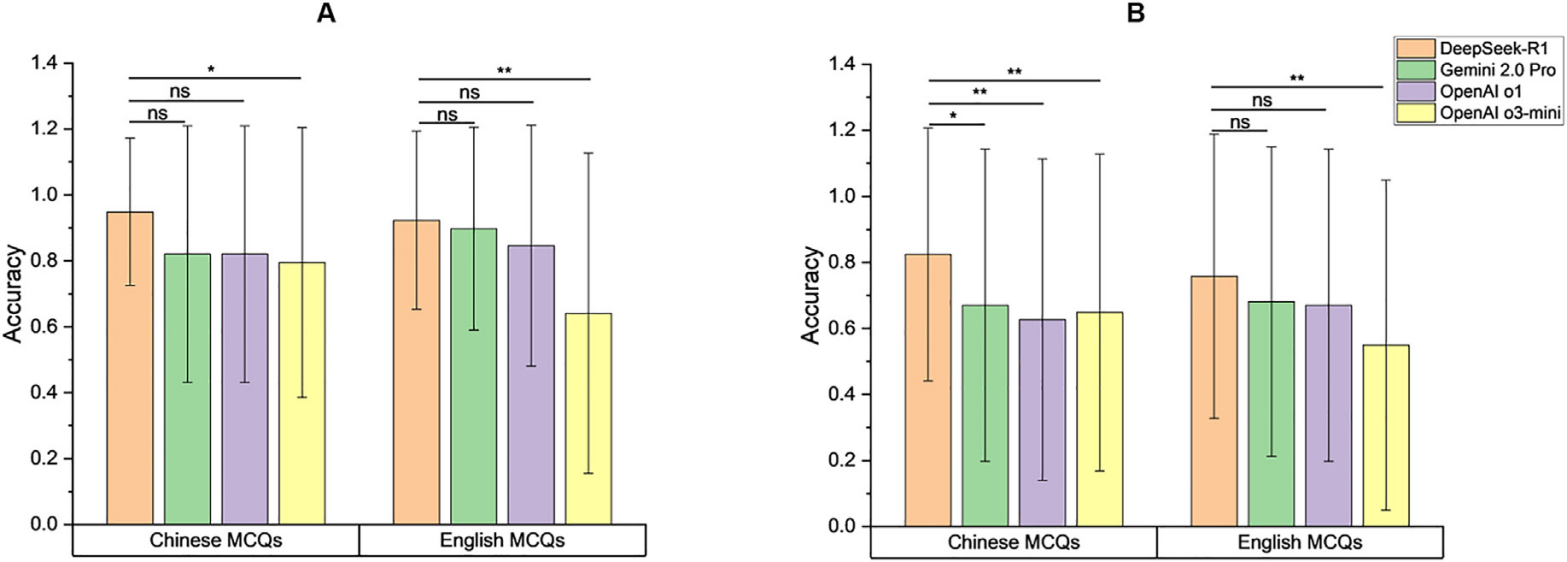
Grouped bar charts showing the comparative performance of four LLMs in reasoning (A) diagnostic and (B) management questions.

As shown in Fig. 3A, DeepSeek-R1 exhibits superior performance compared to OpenAI o3-mini and achieves comparable results to Gemini 2.0 Pro and OpenAI o1 in bilingual diagnostic questions. Besides, in management questions conducted in Chinese, DeepSeek-R1 outperforms the three other LLMs, with all comparisons reaching statistical significance (Fig. 3B).

### 3.4 Reasoning logic analysis

All four LLMs correctly answered the same 63 questions in both Chinese and English MCQs. An example is provided in Supplemental Table 4. All models exhibited similar reasoning logic. First, they identified a history of herpes zoster as the most critical clue, recognizing it as a known causative factor of acute retinal necrosis (ARN). Second, they analyzed key positive clinical signs supporting the diagnosis of ARN. Finally, they systematically ruled out incorrect options by eliminating alternative diagnoses.

There were four questions that only DeepSeek-R1 answered correctly in both Chinese and English MCQs. An example is presented in Supplemental Table 5. Although DeepSeek-R1, Gemini 2.0 Pro, and OpenAI o1 recognized that the key to this question was differentiating between an inflammatory pseudotumor of the lacrimal gland and acute dacryoadenitis, their diagnostic approaches differed. DeepSeek-R1 selected blood routine test and ocular ultrasound, whereas Gemini 2.0 Pro and OpenAI o1 opted for magnetic resonance imaging (MRI). In contrast, OpenAI o3-mini deviated by initially focusing on the exclusion of Graves' ophthalmopathy.

### 3.5 Reasoning error analysis

**Fig. 4:**
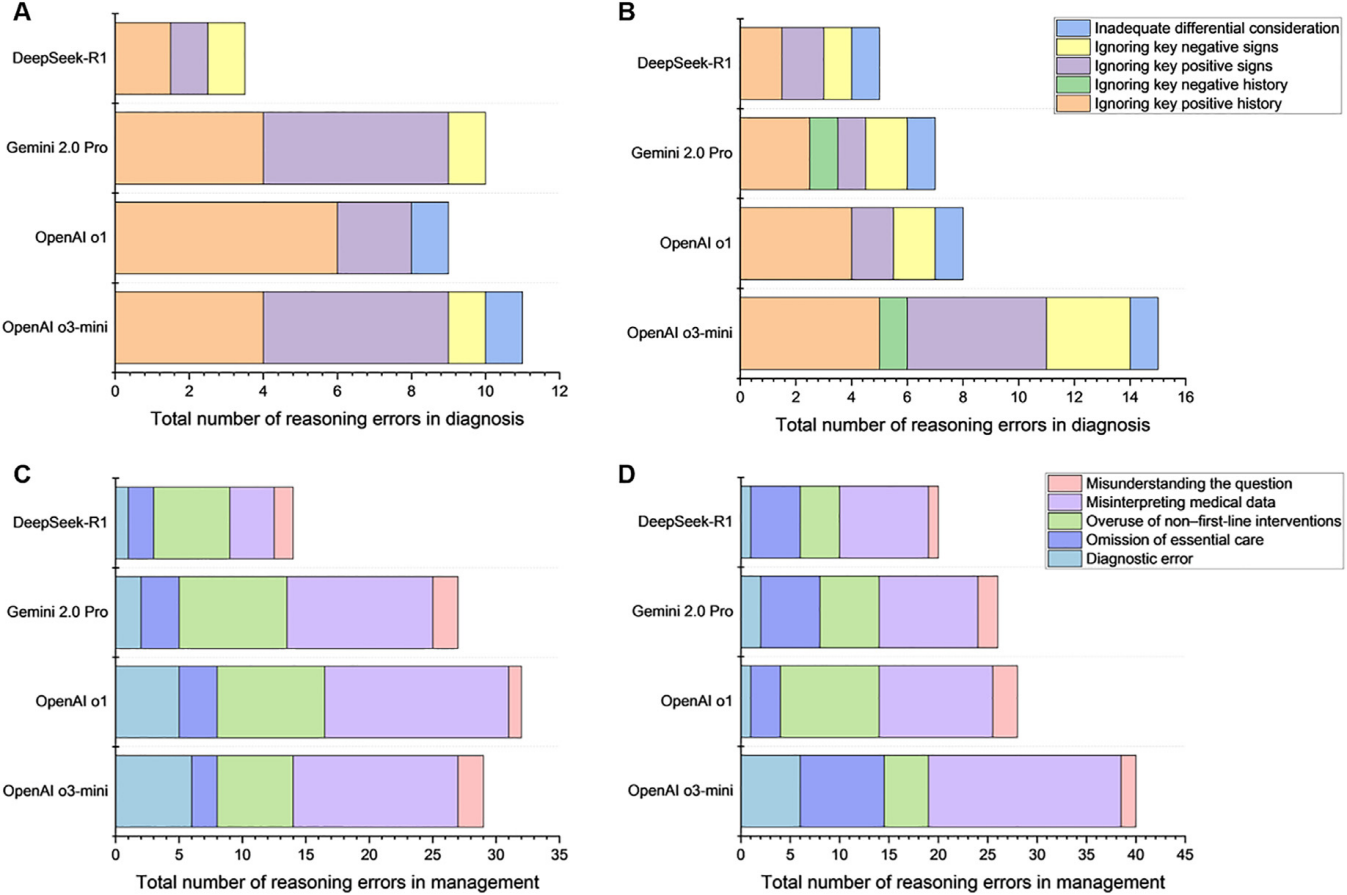
Stacked bar chart illustrating the underlying reasons for reasoning errors in LLMs across different question types. (A) diagnostic questions in Chinese multiple-choice questions (MCQs), (B) diagnostic questions in English MCQs, (C) management questions in Chinese MCQs, and (D) management questions in English MCQs. Error categorizations represent the weighted average assessments from two independent graders.

The Cohen's kappa of two ophthalmologists in analyzing reasoning errors was 0.885. Diagnostic errors resulting from ignoring key positive history and positive signs ranked the top-2 across all LLMs in both Chinese and English diagnostic questions (Fig. 4A and B). In bilingual management questions, misinterpretation of medical data and overuse of non-first-line interventions were the two most common errors across all LLMs (Fig. 4C and D).

## 4 Discussion

Compared to the three other SOTA LLMs, DeepSeek-R1 achieved the best performance in Chinese complex ophthalmology reasoning tasks and performed comparably to Gemini 2.0 Pro in English. DeepSeek-R1 also had the highest number of topics with the highest accuracy and excelled in management questions conducted in Chinese. Reasoning ability analysis showed that the four LLMs shared similar reasoning logic. Ignoring key positive history, ignoring key positive signs, misinterpretation of medical data, and overuse of non-first-line interventions were the most common causes of reasoning errors across all LLMs.

In this study, DeepSeek-R1 demonstrated excellent performance in bilingual complex ophthalmology reasoning tasks, which may be attributed to its innovative training methodology. The DeepSeek team incorporated thousands of high-quality Chain-of-Thought (CoT) data as cold-start data. They observed that fine-tuning the model with cold-start data at the initial phase of reinforcement learning (RL) significantly improved the readability of its output.^17^ Additional strategies, including reasoning-oriented RL, rejection sampling, and supervised fine-tuning, were also implemented in DeepSeek-R1. It is likely that this innovative training approach has enabled DeepSeek-R1 to excel in reasoning tasks such as those in this dataset, which require complex and extended logical chains. Several preprint studies have compared the performance of DeepSeek-R1 and other LLMs in medical contexts. For instance, Mondillo et al. reported that OpenAI o1 outperformed DeepSeek-R1 in pediatric MCQs, achieving a diagnostic accuracy of 92.8% compared to 87.0%.^21^ Zhou et al. found that DeepSeek-R1 generated more readable responses than ChatGPT-4o in patient education materials for spinal surgeries.^22^ Mikhail et al. observed that DeepSeek-R1 and OpenAI o1 demonstrated comparable performance on an English ophthalmology MCQ dataset collected from StatPearls.^23^ However, the questions in their dataset consist of one correct answer and three distractor options, making them less challenging than those in our dataset. Furthermore, as their dataset is publicly available, it may have been utilized in the pre-training or fine-tuning of one or more models. To the best of our knowledge, this is the first study to evaluate bilingual complex ophthalmology reasoning performance across DeepSeek-R1 and three other SOTA LLMs.

DeepSeek-R1 performed slightly better in Chinese than in English on our MCQs, which may be attributed to its higher proportion of Chinese pretraining data. Although DeepSeek-R1 has not disclosed the exact proportion of Chinese and English data, its earlier version, DeepSeek-V2, contained 1.12 times more Chinese tokens than English tokens.^24^ Gemini 2.0 Pro and OpenAI o1 exhibited superior performance in English, a finding consistent with previous research.^25^^,^^26^ However, the reason for OpenAI o3-mini's poorer performance in English MCQs remains unclear. In this study, the accuracy of OpenAI o1 was slightly lower than reported in previous studies,^23^^,^^27^ which may be attributed to the higher difficulty level of the questions used. For instance, in the topic of retinal diseases, where all models demonstrated suboptimal performance, the 11 MCQs included varying numbers of answer choices: 3 questions had five options, 3 had six options, and 5 had eight options. Additionally, 4 questions had more than two correct answers, further increasing the complexity of the task.

All models demonstrated strong reasoning abilities and exhibited similar analytical logic in ophthalmology case analysis. For instance, in diagnostic questions, they first summarize the medical history to identify key clues. Next, they highlight critical positive clinical signs and integrate these with the medical history to formulate a preliminary diagnosis along with a rationale. They then conduct a differential diagnosis for each option, systematically analyzing both supporting and non-supporting factors. Finally, they determine the most appropriate final answer (Supplemental Fig. 1). This logical reasoning sequence in case analysis closely aligns with the diagnostic approach used by human physicians. Even in cases where errors in reasoning occurred, they were not due to fundamental flaws in logical structure but rather a failure to identify the most critical clue or select the most appropriate method. For example, as shown in Supplemental Table 5, DeepSeek-R1, Gemini 2.0 Pro, and OpenAI o1 correctly recognized that the key to the question was differentiating between an inflammatory pseudotumor of the lacrimal gland and acute dacryoadenitis. Although the reference answer aligns with DeepSeek-R1's choice of blood routine tests and ocular ultrasound, it is undeniable that the MRI selected by Gemini 2.0 Pro and OpenAI o1 can also effectively differentiate between the two conditions, but its higher cost prevents it from being the first-line choice.^28^

Ignoring key positive history and positive signs was found to be the top two sources of reasoning error. Interestingly, this result aligns with the causes of diagnostic errors observed in human clinicians.^29^ Misinterpretation of medical data and overuse of non-first-line interventions may result from wrong information in the training data,^30^ variations in reference standards, or the dynamic evolution of medical guidelines. For example, when answering the diagnostic criteria for dry eye using Schirmer's test, the four LLMs failed to reach a consistent conclusion on whether the threshold should be a filter paper wetting length of less than 10 ​mm or less than 5 ​mm within 5 ​min, which may be related to changes in the diagnostic standards for dry eye.^31^^,^^32^

This study has several limitations. First, similar to other studies comparing the performance of different LLMs in ophthalmology,^16^^,^^23^^,^^33^ the MCQs used in this study were published before the models' knowledge cutoff date, making it impossible to ensure that these questions were not included in the models' training data. However, the questions were sourced from VIP documents, and Baidu Wenku has implemented various anti-crawling measures for such documents, including asynchronous loading and data encryption, which reduce the likelihood of these documents being included in the training data. Second, English MCQs were initially translated by DeepSeek-R1 and manually reviewed for accuracy. While this may introduce bias, standardized inputs ensured fair comparison. Future studies could employ third-party translation models to minimize potential bias and evaluate each LLM's full pipeline performance, including both translation and reasoning. Third, the causes of reasoning errors were manually analyzed based on established clinical practice guidelines and consensus statements, differing opinions may exist across different healthcare settings. Finally, due to the lack of reference answers for the reasoning process, we did not quantify the models' reasoning ability. In studies where human answers serve as reference reasoning processes, emerging metrics such as consistency (invariance to logically equivalent inputs), generalization (performance on out-of-distribution data), and explainability (clarity of reasoning steps) can be measured.^34^ Although deductive reasoning, inductive reasoning, abductive reasoning, and analogical reasoning are all crucial to the reasoning capabilities of LLMs, abductive reasoning is more commonly used in the medical domain.^35^ Therefore, future medical research could prioritize the evaluation of this specific ability.

## 5 Conclusions

In summary, compared to the three other LLMs, DeepSeek-R1 exhibited the best performance in bilingual complex ophthalmology reasoning tasks. Although its direct application in clinical practice remains challenging, it holds significant potential for assisting in diagnosis and supporting clinical decision-making.

## Study approval

Not Applicable.

## Author contributions

The authors confirm contribution to the paper as follows: PX and DS designed the study; PX collected the data; PX performed the experiments, PX and YW analyzed and interpreted the data; PX drafted the manuscript; YW, KJ, XC, MH, and DS revised the manuscript. All authors reviewed the results and approved the final version of the manuscript.

## Data availability statement

Data used in this study are available at https://figshare.com/s/cffbc8f89d7032dd13a4.

## Funding

This study was supported by the Global STEM Professorship Scheme (P0046113) and the Start-up Fund for RAPs under the Strategic Hiring Scheme (P0048623) from HKSAR.

## Declaration of competing interest

The authors declare that they have no known competing financial interests or personal relationships that could have appeared to influence the work reported in this paper.
