# Supplementary material for: DeepSeek-R1 outperforms Gemini 2.0 Pro, OpenAI o1, and o3-mini in bilingual complex ophthalmology reasoning
Source: Adv Ophthalmol Pract Res. 2025 May 9;5(3):189–95. doi: 10.1016/j.aopr.2025.05.001 (PMC12269606; doi:10.1016/j.aopr.2025.05.001)
Supplement: Multimedia component 1 [file mmc1.pptx]

## Slide 1
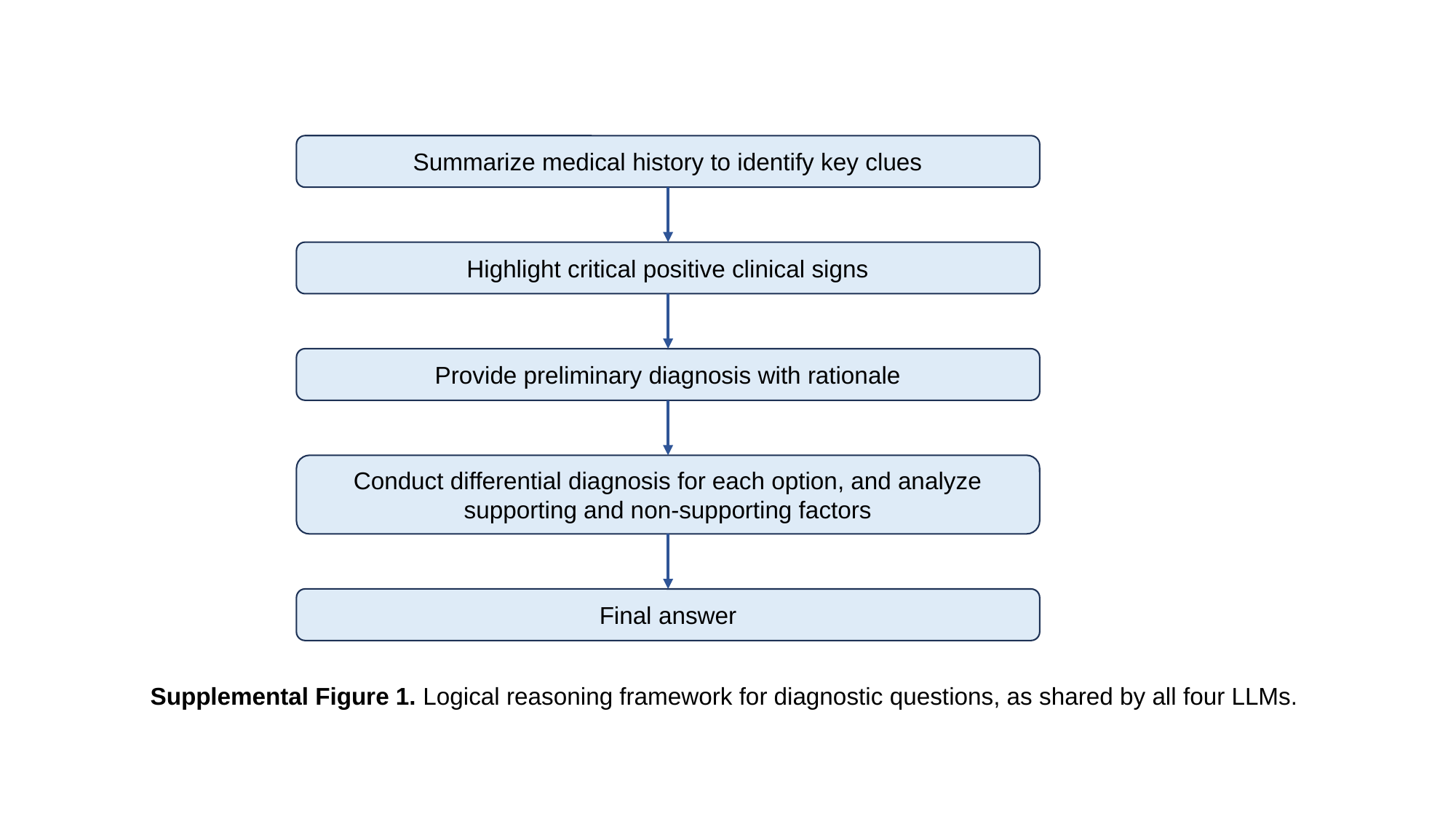

Summarize medical history to identify key clues
Highlight critical positive clinical signs
Provide preliminary diagnosis with rationale
Conduct differential diagnosis for each option, and analyze supporting and non-supporting factors
Final answer
Supplemental Figure 1. Logical reasoning framework for diagnostic questions, as shared by all four LLMs.
