# Supplementary material for: DeepSeek-R1 outperforms Gemini 2.0 Pro, OpenAI o1, and o3-mini in bilingual complex ophthalmology reasoning
Source: Adv Ophthalmol Pract Res. 2025 May 9;5(3):189–95. doi: 10.1016/j.aopr.2025.05.001 (PMC12269606; doi:10.1016/j.aopr.2025.05.001)
Supplement: Multimedia component 2 [file mmc2.docx]

Supplemental Table 1 | Prompts used for translating Chinese ophthalmology multiple-choice question into English (A), generating responses from Large Language Models in Chinese (B) and English (C).

| List | Prompt |
| --- | --- |
| A | Please translate the following Chinese ophthalmology multiple-choice question into English. The requirements are as follows:   1. The translation must maintain professionalism, ensuring that medical terminology and specialized terms are accurate and precise. 2. Keep the format and structure of the original options unchanged. 3. If the question includes specific clinical or ophthalmological background descriptions, ensure that the translated statements are equally rigorous and fluent in English. 4. For any phrases that may cause ambiguity, please express them clearly and professionally in English. Please translate the following question: {question}. |
| B | 你是一个专业的眼科医生，请分析以下病例，并从给定的选项中选出正确答案：{question}. |
| C | You are an expert in ophthalmology. Please analyze the following case and select the correct answer(s) from the given options: {question}. |

Supplemental Table 2 | Comparison of the overall performance of 4 LLMs in Chinese and English MCQs.

| Models | Accuracy | | P value |
| --- | --- | --- | --- |
|  | Chinese MCQs | English MCQs |  |
| DeepSeek-R1 | 0.862 | 0.808 | 0.092 |
| Gemini 2.0 Pro | 0.715 | 0.746 | 0.585 |
| OpenAI o1 | 0.685 | 0.723 | 0.473 |
| OpenAI o3-mini | 0.692 | 0.577 | 0.017* |

* Denotes statistically significant.

Supplemental Table 3 | Subgroup analysis of DeepSeek-R1 and three other LLMs’ performance in different topics. (A) Chinese MCQs, (B) English MCQs.

A

| Topic | Models | | | |
| --- | --- | --- | --- | --- |
|  | DeepSeek-R1 | Gemini 2.0 Pro | OpenAI o1 | OpenAI o3-mini |
| Anterior Segment Diseases | 0.840  (0.639, 0.955) | 0.640  (0.425, 0.820) | 0.600  (0.387, 0.789) | 0.640  (0.425, 0.820) |
| External Eye/Orbital Diseases | 0.875  (0.676, 0.973) | 0.792  (0.578, 0.929) | 0.792  (0.578, 0.929) | 0.750  (0.533, 0.902) |
| Glaucoma | 0.952  (0.762, 0.999) | 0.619  (0.384, 0.819)* | 0.667  (0.430, 0.854) | 0.762  (0.500, 0.839) |
| Ocular Trauma | 0.843  (0.672, 0.947) | 0.688  (0.500, 0.839) | 0.688  (0.500, 0.839) | 0.688  (0.500, 0.839) |
| Refractive Disorders/Strabismus | 0.941  (0.713, 0.999) | 0.882  (0.636, 0.985) | 0.764  (0.501, 0.932) | 0.647  (0.383, 0.858) |
| Retinal Diseases | 0.636  (0.308, 0.891) | 0.727  (0.390, 0.940) | 0.545  (0.234, 0.833) | 0.636  (0.308, 0.891) |

B

| Topic | Models | | | |
| --- | --- | --- | --- | --- |
|  | DeepSeek-R1 | Gemini 2.0 Pro | OpenAI o1 | OpenAI o3-mini |
| Anterior Segment Diseases | 0.720  (0.506, 0.879) | 0.720  (0.506, 0.879) | 0.680  (0.465, 0.851) | 0.560  (0.349, 0.756) |
| External Eye/Orbital Diseases | 0.875  (0.676, 0.973) | 0.667  (0.447, 0.844) | 0.792  (0.578, 0.929) | 0.708  (0.489, 0.874) |
| Glaucoma | 0.905  (0.696, 0.988) | 0.810  (0.581, 0.946) | 0.667  (0.430, 0.854) | 0.524*  (0.298, 0.743) |
| Ocular Trauma | 0.781  (0.600, 0.907) | 0.719  (0.533, 0.863) | 0.656  (0.468, 0.814) | 0.563*  (0.377, 0.736) |
| Refractive Disorders/Strabismus | 0.941  (0.713, 0.999) | 0.941  (0.713, 0.999) | 0.941  (0.713, 0.999) | 0.765  (0.501, 0.932) |
| Retinal Diseases | 0.545  (0.234, 0.833) | 0.636  (0.308, 0.891) | 0.636  (0.308, 0.891) | 0.182  (0.023, 0.518) |

* Denotes statistically significant for comparisons against DeepSeek-R1’s performance. Data were shown with accuracy (95% confidence interval).

Supplemental Table 4 | Selected examples where all models responded correctly to the same question.

| **Question** | Female, 32 years old. Complains of redness, pain, and blurred vision in the right eye for 1 week. History of herpes zoster (lumbar region) 1 month prior.  Ophthalmic Examination:  - Visual acuity: Right eye 0.4 (uncorrected); Left eye 1.2.  - Right eye findings:  - Ciliary congestion.  - Mutton-fat keratic precipitates (KP) on corneal endothelium.  - Tydall’s phenomenon (positive).  - Pupils isochoric and round; lens clear.  - Moderate inflammatory cells present in the vitreous.  - Fundus examination:  - Mild optic disc hyperemia with distinct margins.  - Mild retinal edema.  - Attenuated retinal arteries; peripheral and mid-peripheral arteries exhibit white thread-like appearance with sheathing.  - Patchy yellow-white exudates and scattered dot/patchy hemorrhages in the peripheral retina.  [Multiple-choice Questions]  The most likely diagnosis for this patient is: |
| --- | --- |
| **Options** | A. Behcet's syndrome  B. Acute retinal necrosis **(CORRECT)**  C. Toxoplasmic chorioretinitis  D. Panuveitis  E. Pars planitis  F. Collagen vascular disease |
| **Response (Deepseek R1)** | The most likely diagnosis for this patient is **B. Acute retinal necrosis (ARN)**.  ### Key Analysis:  1. **History of Herpes Zoster (VZV):**  The patient’s recent herpes zoster infection (VZV) strongly suggests a viral etiology. ARN is a rapidly progressive necrotizing retinitis most commonly caused by herpesviruses, including VZV, HSV-1, and HSV-2.  2. **Ophthalmic Findings Supporting ARN:**  - **Anterior Uveitis:** Ciliary congestion, mutton-fat keratic precipitates (KPs), and Tydall’s phenomenon indicate anterior chamber inflammation.  - **Vitritis:** Moderate inflammatory cells in the vitreous suggest posterior segment involvement.  - **Retinal Pathology:**  - Peripheral retinal necrosis (evidenced by patchy yellow-white exudates and hemorrhages).  - Retinal vasculitis (attenuated arteries with white thread-like sheathing).  - Optic disc hyperemia and retinal edema.  These findings align with the classic triad of ARN: **necrotizing retinitis, occlusive vasculitis, and vitritis**.  3. **Distinguishing from Other Options:**  - **A. Behcet’s Syndrome:** Typically presents with oral/genital ulcers, hypopyon, and retinal vasculitis, none of which are mentioned here.  - **C. Toxoplasmic Chorioretinitis:** Usually manifests as focal retinitis adjacent to old scars, not diffuse peripheral necrosis.  - **D. Panuveitis:** While the patient does have panuveitis (inflammation in anterior, intermediate, and posterior segments), this is a descriptive term rather than a specific diagnosis. ARN is the underlying cause of the panuveitis in this case.  - **E. Pars Planitis:** Characterized by intermediate uveitis with snowbanking, absent here.  - **F. Collagen Vascular Disease:** Retinal vasculitis can occur in lupus or other autoimmune diseases, but the history of VZV makes ARN more likely.  ### Conclusion:  The clinical presentation, fundus findings, and recent VZV infection strongly point to **B. Acute Retinal Necrosis** as the specific diagnosis. While panuveitis (D) is present, ARN is the definitive etiology driving the inflammation.  **Answer:** B. Acute retinal necrosis |
| **Response**  **(Gemini 2.0 PRO)** | Let's break down this case step-by-step, considering the key findings and differential diagnoses:  **Key Findings:**  * **Recent Herpes Zoster:** This is the most crucial piece of information. The history of herpes zoster (shingles) one month prior, even in the lumbar region, strongly suggests a varicella-zoster virus (VZV) etiology.  * **Unilateral Involvement:** The symptoms and signs are confined to the right eye.  * **Anterior Segment Inflammation:**  * Ciliary congestion: Indicates anterior segment inflammation (uveitis).  * Mutton-fat KPs: These are large, greasy-appearing precipitates on the corneal endothelium, characteristic of *granulomatous* uveitis. This is a very important clue.  * Tyndall's phenomenon (positive): Indicates the presence of inflammatory cells in the anterior chamber, confirming anterior uveitis.  * **Posterior Segment Inflammation:**  * Inflammatory cells in the vitreous: Indicates vitritis.  * Mild optic disc hyperemia: Suggests optic nerve involvement (but the margins are distinct, ruling out severe optic neuritis).  * Mild retinal edema.  * Attenuated retinal arteries with sheathing: This is a very significant finding, pointing towards retinal vasculitis.  * Peripheral yellow-white exudates and hemorrhages: These are indicative of retinal necrosis and inflammation. The *peripheral* location is very important.  *Reduced VA: Vision is significantly reduced in the effected eye.  **Differential Diagnosis Analysis:**  * **A. Behcet's Syndrome:** Behcet's can cause panuveitis, but it's usually bilateral, recurrent, and often associated with oral and genital ulcers. The mutton-fat KPs and strong history of recent zoster make this less likely. Behcet's *can* cause retinal vasculitis, but the other features don't fit as well.  * **B. Acute Retinal Necrosis (ARN):** This is the *most likely* diagnosis. ARN is a severe, necrotizing retinitis most commonly caused by VZV (or HSV). The key features that point to ARN are:  * **History of Herpes Zoster:** ARN is often (but not always) seen in patients with a prior history of zoster or chickenpox.  * **Granulomatous Anterior Uveitis:** The mutton-fat KPs suggest a granulomatous inflammatory process, which is consistent with viral uveitis.  * **Retinal Vasculitis:** The attenuated, sheathed arteries are a hallmark of ARN.  * **Peripheral Retinal Necrosis:** The peripheral yellow-white exudates and hemorrhages represent areas of retinal necrosis. The *peripheral* location is classic for ARN, which typically starts in the periphery and progresses centrally.  * **Vitritis:** The presence of inflammatory cells in the vitreous is common in ARN.  * **C. Toxoplasmic Chorioretinitis:** Toxoplasmosis typically presents with a focal, well-defined area of retinochoroiditis ("headlight in the fog" appearance due to vitritis overlying the lesion). It *can* cause KPs, but mutton-fat KPs are less typical. The history of zoster and the pattern of peripheral necrosis with vasculitis don't fit toxoplasmosis.  * **D. Panuveitis:** While the patient *does* have panuveitis (inflammation of the anterior uvea, vitreous, and retina), this is a descriptive term, not a specific diagnosis. We need to identify the *cause* of the panuveitis.  * **E. Pars Planitis:** Pars planitis (intermediate uveitis) is characterized by inflammation primarily in the vitreous and peripheral retina, often with "snowbanking" over the pars plana. While vitritis is present, the presence of mutton-fat KPs, retinal vasculitis, and the history of zoster point away from pars planitis. Pars planitis is also usually bilateral.  * **F. Collagen Vascular Disease:** While collagen vascular diseases (like lupus or rheumatoid arthritis) *can* cause uveitis and retinal vasculitis, they are much less likely given the acute onset, unilateral presentation, and strong history of recent herpes zoster. The mutton-fat KPs also argue against this.  **Conclusion:**  Based on the clinical presentation, the history of recent herpes zoster, the presence of granulomatous anterior uveitis, retinal vasculitis, and peripheral retinal necrosis, the most likely diagnosis is **Acute Retinal Necrosis (ARN)** due to reactivation of varicella-zoster virus.  **Final Answer:** The correct answer is **B. Acute retinal necrosis**. |
| **Response (OPENAI o1)** | The most likely diagnosis for this patient is **B. Acute Retinal Necrosis (ARN)**.  **Explanation:**  **Key Clinical Findings:**  1. **History of Herpes Zoster:** The patient had herpes zoster (shingles) in the lumbar region one month prior. Varicella-zoster virus (VZV), which causes shingles, is a known causative agent of ARN.  2. **Ophthalmic Signs:**  - **Anterior Segment Inflammation:** Ciliary congestion, mutton-fat keratic precipitates, positive Tyndall's phenomenon indicate anterior uveitis.  - **Posterior Segment Involvement:**  - **Vitreous Inflammation:** Moderate inflammatory cells in the vitreous.  - **Retinal Findings:**  - **Peripheral Retinal Necrosis:** Patchy yellow-white exudates in the peripheral retina.  - **Retinal Vasculitis:** Attenuated retinal arteries with white thread-like sheathing.  - **Retinal Hemorrhages:** Scattered dot/patchy hemorrhages in the peripheral retina.  - **Optic Disc Hyperemia and Mild Retinal Edema:** Indicate posterior segment inflammation.  3. **Unilateral Presentation:** ARN typically presents unilaterally, especially in immunocompetent individuals.  **Why Acute Retinal Necrosis is the Best Fit:**  - **Causative Agents:** ARN is commonly caused by herpes viruses, particularly VZV and HSV.  - **Clinical Features of ARN:**  - Rapidly progressive, necrotizing retinitis starting in the peripheral retina.  - Prominent vitritis (inflammatory cells in the vitreous).  - Occlusive retinal vasculitis (arterial sheathing).  - Optic nerve involvement and uveitis.  - **Temporal Relationship:** The onset of ocular symptoms one month after a herpes zoster outbreak aligns with the typical progression of VZV reactivation leading to ARN.  **Other Options Considered:**  - **A. Behçet's Syndrome:** Usually presents with recurrent oral and genital ulcers and bilateral ocular involvement. Retinal vasculitis can occur but is less likely given this patient's history and unilateral findings.    - **C. Toxoplasmic Chorioretinitis:** Typically presents with focal necrotizing retinochoroiditis ("headlights in fog") and is often associated with congenital infection or immunosuppression.  - **D. Panuveitis:** While the patient has both anterior and posterior segment inflammation, panuveitis is a broad term. ARN is a specific diagnosis that explains all findings.  - **E. Pars Planitis:** A type of intermediate uveitis affecting the vitreous and peripheral retina, but usually bilateral and without the severe retinal necrosis seen in ARN.  - **F. Collagen Vascular Disease:** Can cause retinal vasculitis but is less likely without systemic symptoms and given the recent VZV infection.  **Conclusion:**  Given the patient's recent VZV infection, unilateral ocular findings consistent with retinal necrosis, vasculitis, and both anterior and posterior segment inflammation, **Acute Retinal Necrosis** is the most probable diagnosis.  **Answer:** **B. Acute retinal necrosis** |
| **Response (OPENAI o3-mini)** | Based on the given case details, the most likely diagnosis is **B. Acute retinal necrosis**.  Here’s the reasoning:  - The patient has a history of herpes zoster, which is a known risk factor for developing **acute retinal necrosis (ARN)**. ARN typically occurs in immunocompetent patients with a recent history of viral infection, especially herpes zoster or herpes simplex virus.  - The key findings, such as **mutton-fat keratic precipitates**, **positive Tydall's phenomenon**, and **vitreous inflammation**, point to an intraocular inflammation (uveitis).  - The **retinal changes**, such as **white thread-like sheathing of the arteries**, **patchy yellow-white exudates**, and **dot/patchy hemorrhages** in the peripheral retina, are highly suggestive of **retinal necrosis** associated with viral infections like herpes zoster.  - ARN can cause significant vision loss if not treated promptly.  Other options:  - **A. Behcet's syndrome**: Behcet's disease is a multi-system inflammatory disorder, but the specific retinal findings are not typical of this condition.  - **C. Toxoplasmic chorioretinitis**: This usually presents with more localized retinal lesions and is associated with immunocompromised states.  - **D. Panuveitis**: While panuveitis could cause similar symptoms, the retinal findings and the herpes zoster history make ARN more likely.  - **E. Pars planitis**: This typically affects younger individuals and is associated with non-infectious inflammation of the peripheral retina, which does not fit the case.  - **F. Collagen vascular disease**: This could cause uveitis but would likely involve other systemic symptoms that are not present here.  Thus, **acute retinal necrosis (ARN)** is the most likely diagnosis. |

Supplemental Table 5 | Selected examples where only Deepseek R1 responded correctly to the same question.

| **Question** | Female, 45 years old, presents with bilateral eyelid swelling and orbital pain for half a month. Examination findings: Visual acuity OD 1.0, OS 1.0. Both upper eyelids exhibit an S-shaped curvature with erythematous and swollen skin. Tenderness localized to the lateral upper eyelids. Swollen palpebral lacrimal glands observed in the superotemporal fornix. Lateral bulbar conjunctival congestion and edema (+). Corneas transparent. Fundus examination unremarkable. Vital signs: temperature 36.5°C, blood pressure 130/80 mmHg. History of recent cold. No enlargement of superficial lymph nodes.  [Multiple-choice Question] Further investigations required for definitive diagnosis include: |
| --- | --- |
| **Options** | A. Blood routine test + ocular ultrasound **(CORRECT)**  B. Orbital CT  C. MRI  D. Thyroid function tests  E. Blood routine test |
| **Response (Deepseek R1)** | The patient's presentation of bilateral S-shaped eyelid curvature, erythema, tenderness over the lateral upper eyelids, and swollen palpebral lacrimal glands in the superotemporal fornix strongly suggests acute dacryoadenitis. The bilateral involvement and recent viral illness point to a potential viral etiology, though bacterial or systemic inflammatory causes (e.g., sarcoidosis, IgG4-related disease) should also be considered. Key investigations should aim to confirm the diagnosis and identify the underlying cause.  Blood Routine Test: Essential to assess for leukocytosis (indicative of bacterial infection) or lymphocytosis (suggesting viral etiology). Inflammatory markers (e.g., ESR, CRP) may support systemic inflammation.  Ocular Ultrasound: A non-invasive, first-line imaging modality to evaluate the lacrimal gland for enlargement, echotexture changes, and rule out abscess formation. It is particularly effective for superficial structures like the palpebral lacrimal gland, which is already clinically evident.  Orbital CT/MRI: While CT provides detailed orbital imaging and is superior for detecting deeper orbital involvement or abscesses, it involves radiation exposure. MRI offers superior soft-tissue resolution but is less accessible. Given the localized findings and absence of deeper orbital signs (e.g., proptosis, optic nerve compromise), ultrasound suffices for initial evaluation.  Thyroid Function Tests: Less relevant here, as there are no signs of thyroid eye disease (e.g., proptosis, lid retraction, EOM restriction).  Conclusion: The combination of blood routine test (to identify infection/inflammation) and ocular ultrasound (to confirm lacrimal gland pathology) provides the most appropriate and efficient initial investigations for definitive diagnosis. Orbital CT/MRI may be reserved for atypical or refractory cases.  Answer: A. Blood routine test + ocular ultrasound |
| **Response**  **(Gemini 2.0 PRO)** | Let's analyze this case carefully to determine the best investigations for a definitive diagnosis.  **Key Findings and Differential Diagnosis:**  * **Bilateral eyelid swelling, orbital pain, S-shaped curvature of upper eyelids, erythematous and swollen skin, tenderness localized to the lateral upper eyelids, swollen palpebral lacrimal glands:** These strongly suggest inflammation of the lacrimal glands (dacryoadenitis). The bilateral presentation is crucial. The S-shaped curve is characteristic of lacrimal gland enlargement.  * **Lateral bulbar conjunctival congestion and edema (+):** Consistent with adjacent inflammation.  * **Normal visual acuity, transparent corneas, unremarkable fundus:** Rules out significant intraocular involvement like uveitis, optic neuritis, or other conditions directly affecting the retina or optic nerve. This makes acute, severe infections impacting vision less likely.  * **Recent cold:** A viral upper respiratory tract infection (URTI) can be a trigger for certain types of dacryoadenitis.  * **No lymphadenopathy:** This makes a widespread, systemic infectious process less likely, but doesn't rule it out completely.  *Normal Temperature: Not Febrile.  Given this clinical picture, the primary differential diagnoses include:  1. **Idiopathic Orbital Inflammation (IOI), formerly known as Orbital Pseudotumor, specifically dacryoadenitis:** This is a diagnosis of exclusion. It's a non-specific inflammatory condition that can affect any part of the orbit, including the lacrimal gland.  2. **Infectious Dacryoadenitis:** This could be viral (especially given the history of a recent cold) or, less likely given the lack of fever and bilateral presentation, bacterial.  3. **Systemic Inflammatory Diseases:** Conditions like sarcoidosis, granulomatosis with polyangiitis (GPA, formerly Wegener's), IgG4-related disease, and rarely, thyroid eye disease (though less typical without other thyroid eye signs) can cause dacryoadenitis.  4. Other very rare possibilities (lymphoma, etc).  **Why each option is correct/incorrect:**  * **A. Blood routine test + ocular ultrasound:** A blood routine test (specifically a complete blood count, CBC, and erythrocyte sedimentation rate, ESR, or C-reactive protein, CRP) is essential to assess for general inflammation and rule out obvious infection. Ocular ultrasound can be useful to visualize the lacrimal gland and assess for abscess formation, but it's not the *most* important next step for *definitive* diagnosis. It would be supportive, but not definitive.  * **B. Orbital CT:** A CT scan is a good imaging modality, but *MRI is superior for soft tissue detail.* CT is better for bony detail. While CT can show lacrimal gland enlargement, it may not differentiate the underlying cause as well as MRI.  * **C. MRI:** **This is the BEST answer.** An MRI of the orbits *with contrast* is the imaging modality of choice for evaluating dacryoadenitis. It provides excellent soft tissue detail, allowing visualization of the lacrimal gland and surrounding structures. It can help differentiate between inflammatory conditions, infection (though less likely based on presentation), and other masses. Crucially, MRI can better characterize the inflammatory process and help distinguish between IOI, sarcoidosis, and other etiologies than CT. Contrast enhancement patterns are particularly helpful.  * **D. Thyroid function tests:** While thyroid eye disease *can* rarely present with isolated lacrimal gland involvement, it's much less likely given the other, more prominent features of dacryoadenitis. Thyroid function tests (TSH, free T4) should be considered, but they are *not* the *most crucial* initial investigation for reaching a definitive diagnosis *in this specific presentation*. It's part of a broader workup, but not the priority.  * **E. Blood routine test:** As mentioned in (A), a blood routine test (CBC, ESR/CRP) is *essential*, but not *sufficient* for a definitive diagnosis. It helps assess the presence and degree of inflammation and potential infection, but it doesn't pinpoint the *cause* of the dacryoadenitis.  **Conclusion and Definitive Diagnosis Strategy:**  The most important next step for a *definitive* diagnosis is an **MRI of the orbits with contrast (C)**. This is followed closely in importance by a **blood routine test (A, E - but A is better because it includes ultrasound)**. Thyroid function tests (D) are part of the broader workup, but lower priority than MRI and blood work. CT (B) is a reasonable imaging modality but is less sensitive for soft tissue characterization than MRI.  The definitive diagnostic approach would involve:  1. **MRI of the orbits with contrast:** To characterize the lacrimal gland inflammation and rule out other orbital pathology.  2. **Blood tests:** CBC, ESR/CRP, and potentially other tests based on MRI findings and clinical suspicion (e.g., ACE for sarcoidosis, ANCA for GPA, IgG4 levels).  3. **Thyroid function tests:** To rule out thyroid eye disease, even though it's less likely.  4. **Consideration of biopsy:** If the diagnosis remains uncertain after imaging and blood work, a biopsy of the lacrimal gland may be necessary, particularly to rule out malignancy or specific inflammatory conditions.  Therefore, the best single answer from the provided options is **C. MRI**. |
| **Response (OPENAI o1)** | Based on the clinical presentation and examination findings, the patient is exhibiting signs of lacrimal gland enlargement or pathology. The lacrimal gland is located in the superolateral part of the orbit, and its involvement can present with swelling and tenderness in the lateral upper eyelid, as seen in this patient. Bilateral swelling and recent history of a cold suggest a possible inflammatory process, such as acute dacryoadenitis, which can be viral in origin.  To achieve a definitive diagnosis, it is crucial to obtain detailed imaging of the orbital and lacrimal gland structures. While both CT and MRI scans can provide imaging of the orbit, MRI is superior in differentiating soft tissue structures and masses within the orbit due to its high contrast resolution. MRI can help distinguish between inflammatory conditions, neoplastic processes, and other pathologies affecting the lacrimal gland. It can provide detailed images of the lacrimal gland's size, shape, and any masses present, which is essential for accurate diagnosis and appropriate management.  Therefore, the most appropriate investigation for a definitive diagnosis in this case is an MRI.  **Answer:** C. MRI |
| **Response (OPENAI o3-mini)** | The patient's presentation, including bilateral eyelid swelling, orbital pain, erythematous and swollen skin, and tenderness, suggests a possible inflammatory or infectious condition affecting the eyelids and lacrimal glands. Additionally, the presence of swollen palpebral lacrimal glands and lateral bulbar conjunctival congestion could indicate a condition such as dacryoadenitis (inflammation of the lacrimal gland), which can be caused by infections or systemic conditions.  Given the absence of superficial lymph node enlargement, normal visual acuity, and a history of a recent cold, the primary concern seems to be an inflammatory process, potentially related to viral infection (e.g., mumps or an upper respiratory viral infection) or an autoimmune condition. Thyroid disorders, particularly thyroid eye disease, could also present with similar symptoms, especially with orbital involvement.  **Correct answer: D. Thyroid function tests**  Thyroid disease, particularly thyroid eye disease (Graves' orbitopathy), can cause orbital inflammation and eyelid changes. In this case, thyroid function tests are warranted to assess for any underlying thyroid dysfunction that might explain the symptoms. If thyroid disease is ruled out, further imaging may be needed for a definitive diagnosis. |
